# Prospective single center trial of next-generation sequencing analysis in metastatic renal cell cancer: the MORE-TRIAL

**DOI:** 10.4155/fsoa-2017-0150

**Published:** 2018-03-14

**Authors:** Svenja Dieffenbacher, Stefanie Zschäbitz, Luisa Hofer, Gencay Hatiboglu, Weibin Hou, Markus Hohenfellner, Stefan Duensing, Holger Sültmann, Sascha Pahernik, Carsten Grüllich

**Affiliations:** 1 The Renal Cancer Center at the National Center for Tumor Diseases (NCT) Heidelberg, Germany, Department of Urology, Heidelberg University Hospital, Im Neuenheimer Feld 110, Heidelberg, Germany; 2 The Renal Cancer Center at the National Center for Tumor Diseases (NCT) Heidelberg, Germany, Department of Medical Oncology, National Center for Tumor Diseases Heidelberg, Heidelberg University Hospital, Im Neuenheimer Feld 460, Heidelberg, Germany; 3 The Renal Cancer Center at the National Center for Tumor Diseases (NCT) Heidelberg, Germany, Section of Molecular Uro-oncology, Department of Urology, Heidelberg University Hospital, Im Neuenheimer Feld 110, Heidelberg, Germany; 4 The Renal Cancer Center at the National Center for Tumor Diseases (NCT) Heidelberg, Germany, Cancer Genome Research Group, German Cancer Consortium (DKTK), Heidelberg, Germany, German Cancer Research Center (DKFZ) & National Center for Tumor Diseases (NCT), Heidelberg, Germany; 5 The Renal Cancer Center at the National Center for Tumor Diseases (NCT) Heidelberg, Germany, Department of Urology, Nuremberg General Hospital, Paracelsus Medical University, Nuremberg, Germany

**Keywords:** clonal evolution, next-generation sequencing, renal cell carcinoma, targeted therapy, tumor heterogeneity

## Abstract

**Aim:**

Targeted therapies have substantially improved the survival of patients with metastatic clear cell renal cell cancer. No prognostic or predictive biomarkers are available. Comprehensive genetic profiling offers the opportunity to define prognostic and predictive signatures aiming at a more personalized approach to treatment.

**Methods:**

In this prospectively conducted cohort study, tumor tissue and liquid biopsies are sampled at baseline and upon first and second progression under systemic treatment. Samples will be analyzed by whole-exome sequencing to generate prognostic and predictive patterns for systemic therapies.

**Discussion:**

This study is aiming at exploring genetic profiles with prognostic and predictive value in metastatic renal cell cancer patients. Clonal evolution facilitating resistance to systemic treatment will be investigated by repeat biopsies.

## Lay abstract

Kidney cancer (renal cell carcinoma) is genetically diverse between different patients. Further, within a single affected individual, multiple genetic clones develop in the tumor and different metastases. Current treatment standard is molecularly targeted, albeit currently no genetic signatures can predict if an individual will benefit from treatment. This study aims at defining genetic profiles of kidney tumors of individual patients at the start of systemic therapy and at the timepoints of treatment failure. The aim will be to define markers that could help to choose the best therapy at baseline and at tumor progression.

Renal cell carcinoma (RCC) accounts for about 2–3% of all cancers and shows its highest incidence in Western countries [1]. About 75% of RCCs are of the clear-cell subtype. Localized disease is commonly diagnosed as an incidental finding and can be successfully managed with partial nephrectomy or radical nephrectomy, whereas metastatic RCC is known to be refractory to conventional chemotherapy.

Over the last decade, the availability of targeted therapies has led to a substantial improvement in outcome for patients with metastatic RCC [2]. Inhibition of VEGF, inhibition of mTORC1 and more recently, targeted immunotherapy with PD1-inibitition are the available therapeutic options for the treatment of metastatic disease [3]. However, a curative treatment is still not available and the disease finally progresses leading to the death of the patient. The choice of treatment and therapeutic sequencing is still based on all-comer clinical trials with objective response rates around 40%. Molecular biomarkers for prognosis and prediction of clinical benefit are still not available. Moreover, RCC has been demonstrated to be extremely heterogeneous within single patients [4,5] and the molecular mechanisms driving therapy resistance are still scarcely understood. Hence, a better molecular understanding of RCC is urgently needed. With the availability of high-throughput molecular methods, especially next-generation sequencing (NGS), novel tools are in the hands of clinicians and researchers to better explore the genetic landscape of an individual tumor and its susceptibility to molecular targeted therapy as well as the escape mechanism leading to treatment failure and disease progression.

The MORE trial is a prospective biomarker study designed to investigate molecular alterations in patients with metastatic RCC. Whole-exome analysis of tumor samples and circulation tumor DNA will be performed at baseline and upon progression under systemic treatment with approved agents according to the European guidelines of urology by using whole-genome sequencing of primary and biopsy tissue samples as well as liquid biopsies from therapy-naive patients at baseline and after first and second drug treatment.

## Methods

### Study objectives

To investigate the molecular alterations occurring under targeted drug treatment and to learn about drug resistances, baseline liquid biopsies and tissue biopsies will be analyzed and compared with mutational patterns occurring under first- and second-line targeted therapy. Potential molecular targets for personalized therapy of progressive disease should be analyzed to improve patient care and outcome.

The study is approved by the ethics committee at the Heidelberg University Medical Faculty (S-539/2013). The study is listed on clinicaltrials.gov (NCT02208128) and on the National Study Register (DRKS0006193).

A feasibility analysis was performed with four patients before implementation of the full study. Tissue and liquid biopsies generated sufficient DNA for analyzing mutational patterns [6].

Patient selection and study design: this NGS-based trial is a monocenter prospective cohort study with explorative character to investigate molecular alterations under drug treatment in patients with metastatic RCC. Treatment-naive patients with metastatic clear cell RCC are eligible.

The study will include a maximum of 100 patients with metastatic RCC on the assumption of a maximum of 20 patients to be recruited annually within a period of 5 years. The study is open for recruitment and is expected to recruit until 2022. The eligibility criteria are shown in Table 1.

**Table 1.** **Eligibility criteria.**

**Inclusion criteria**

1. Presence of metastatic lesions easily accessible for biopsy

2. Age > 18 years

3. Histopathological subtype of clear cell renal carcinoma

4. Absence of contraindications for approved drug treatment

5. Presence of indication for systemic therapy

**Exclusion criteria**

1. Pre-existing psychological diseases

2. Further malignant diseases

3. Patients with increased bleeding tendency or increased risk for wound healing deficit

4. Absence of legal capacity or patients unable to consent

5. Presence of contraindications for surgical treatment

An average response period of 11 months per drug is assumed according to the literature available [7]. According to the European Association of Urology guidelines [8] and the European Society for Medical Oncology guidelines [9], patients will receive a tumor biopsy of primary tumor tissue or metastatic sites for histopathological approval of clear cell renal carcinoma. If debulking tumor surgery is indicated, patients will undergo a partial or radical nephrectomy, respectively, before starting with systemic first-line drug treatment due to the current standard.

DNA from tumor tissue and a liquid biopsy at baseline, after first and second progression under standard of care drug treatment according to physician's choice will be obtained and subjected to whole-exome sequencing. Progress evaluation is performed according to RECIST and a tumor biopsy will be performed on one accessible progressing lesion in order to obtain vital tumor for DNA preparation.

**Figure 1.**
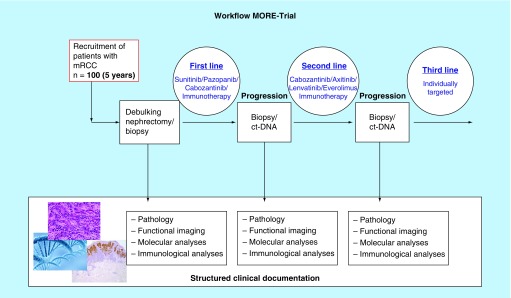
**MORE study diagram and workflow.** ct-DNA: Circulating tumor-DNA; mRCC: Metastatic renal cell carcinoma.

Since there is no clear evidence for a clinical benefit with any approved drug in the third-line situation, the treatment of choice in third line will be based upon the obtained mutational patterns, in other words, the approved targeted drug will be chosen that best covers the pathways altered by detected mutations. This includes immunotherapy which could be related with tumor mutational burden as a potential predictive signature. The MORE study diagram and workflow is shown in Figure 1.

Sample collection: standard treatment of patients with metastatic RCC includes a cytoreductive nephrectomy. Small samples of tumor tissue as well as samples of healthy kidney tissue will be obtained from tumor preparation. In addition to a standard blood sample, 10 ml of blood will be obtained for circulating tumor-DNA (ct-DNA) diagnostics. Processing of blood for ct-DNA collection will be performed as described previously [10]. In brief, blood from patients will be collected in EDTA tubes (Sarstedt, Nümbrecht, Germany) and processed within 1 h. Samples will be centrifuged at 2000  ×*g* for 10  min at 10 °C, and plasma supernatant will be stored at -80 °C until use. DNA extraction will be from 500  μl aliquots of frozen plasma using the QIAamp Blood Mini Kit (Qiagen, Hilden, Germany) according to manufacturer's protocol. Analysis and detection of mutation in ct-DNA will be performed according to previously published algorithms [11,12]. 2 weeks after surgery, systemic therapy according to current guidelines will be initiated.

Isolation, quantification and quality control of genomic DNA, fresh frozen surgical tumor tissue and biopsy samples (10–30 mg) will be mechanically disrupted and homogenized using the TissueRuptor (Qiagen, Hilden, Germany). Genomic DNA will be extracted using the QIAamp DNA Mini Kit (Qiagen) according to the manufacturer's protocol. As germline control, genomic DNA from matched blood cells will be isolated by the QIAamp DNA Blood Mini Kit (Qiagen). DNA concentrations will be determined using the Qubit (Thermo Fisher Scientific, MA, USA), and DNA integrity will be assessed using the TapeStation (Agilent Technologies, CA, USA).

### Library construction & exome sequencing

Sequencing libraries will be prepared from 200 ng genomic DNA. Prior to library preparation, all DNA samples will be sheared to an average fragment length of 150 bp using the Covaris S220 ultrasonicator. Exome-enriched sequencing libraries will be prepared using the Agilent SureSelectXT Human All Exon V5+UTR kit (low input protocol). Library sizes and qualities will be evaluated before and after capture by Bioanalyzer 2100 analysis using the High Sensitivity DNA Kit (Agilent) and quantified using the Qubit dsDNA HS Assay kit (Thermo Fisher Scientific). All libraries will be subjected to 100 bp paired-end sequencing on the Illumina HiSeq 2000 v3 at the DKFZ Genomics and Proteomics Core Facility. Selected variants will further be validated by bidirectional Sanger sequencing on an ABI 3130 Genetic Analyzer (Thermo Fisher Scientific) using the BigDye Terminator v1.1 Cycle Sequencing Kit (Thermo Fisher Scientific) as described previously [13].

### Feasibility data

**Figure 2.**
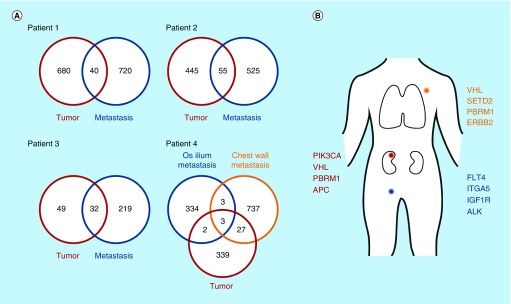
**MORE feasibility data.** **(A)** Number of exclusive and shared mutations identified in primary tumor tissue and metastasis biopsies. **(B)** Functional coding mutations in the primary tumor (kidney) and metastasis biopsies (lymph node, chest wall) from one patient. Reprinted with permission from [6] Dietz S, Sultmann H, Du Y *et al*. (2017).

A small feasibility cohort of four patients was subjected to sequencing of primary and metastasis upon first progression according to the requirements of the funding body (HIPO-POP Heidelberg). The feasibility to obtain sufficient tumor DNA by biopsy for NGS could be demonstrated [6]. We could further show that the tumor evolution under therapy led to a diverse patient-specific mutational pattern with little overlap between primary tumor and progressing metastasis (Figure 2). Feasibility data resulted in the approval of full funding for the MORE study opening the protocol for recruitment.

## Discussion

Clear cell renal carcinoma is a genetically heterogeneous disease. Founding genetic alterations that can be detected in most patients are genetic or epigenetic changes leading to a biallelic loss of function of the *VHL* gene. However, apart from *VHL*, a very diverse intra- and inter-individual genetic heterogeneity has been observed when different regions of single tumors and different metastatic sites have been analyzed [5]. Moreover, upon progression under treatment with everolimus, a further increase in genetic variability has been demonstrated, albeit only in a few individuals [3,5]. The MORE protocol aims at obtaining systematic genomic data from a large prospective cohort treated with current standard of care including targeted therapies and immune-checkpoint inhibitors. Tumor samples at baseline will be obtained either from partial or total nephrectomy or from fine needle biopsies. To detect clonal evolution driven by systemic therapy resulting in resistance, rebiopsies of progressing metastatic lesions or the primary tumor will be performed after first and second progression. All samples will be analyzed by whole-exome sequencing. Mutational signatures will be compared between individuals and interindividuals between baseline and progression. The aim of the study is to detect prognostic patterns between patients as well as mutational signatures capable of response prediction to the selected therapies. Also, mutational patterns that signify a resistance development to a chosen drug after an initial response will be searched for within the genomic data. We already performed a small pilot study to prove the feasibility of this approach. In this small cohort we could detect large variations of functional mutations between individuals and strikingly little overlap between baseline and progression in individual patients including potentially druggable alteration like *BRCA* and *PI3K* mutations. The current standard of care in first line at our center is sunitinib or pazopanib for patients with a reduced performance status. In the near future, immune-checkpoint inhibitors as well as newer tyrosine kinase inhibitors (TKIs) like cabozantinib will likely be approved. In second line, nivolumab is currently the standard of care with rare cases receiving cabozantinib or lenvatinib and everolimus if the clinical situation requires a fast objective response. Hence, to prevent the cohorts with a specified therapy from getting too small we aim at treating larger groups of patients uniformly. Further, analysis of molecular signatures with regard to predictive factors will have to consider comparing groups of different modes of action-targeted agents versus immune-checkpoint inhibitors as well as analyzing TKIs followed by checkpoint-inhibitor and checkpoint-inhibitor followed by TKI sequences, respectively. In later lines of treatment, approved targeted substances will be chosen according to mutations found in the individual patient. The recommendations for a personalized therapy based on genetic findings within this study will be made by our molecular tumor board, taking into consideration the functional relevance as well as the allelic frequencies of detected genomic alterations (at least 20%) and the availability of an approved drug with target specific activity. Clinical follow-up within our study is aiming at the validation of the molecular tumor board recommendation.

Since the analysis of primary tumor or a single metastatic site at baseline or progression may not detect the relevant clone driving progression in other sites, we will obtain liquid biopsies in parallel for detection and mutational analysis of ct-DNA. This relatively new technique may help to avoid the necessity for invasive biopsies in the future [10]. However, it has yet to be shown that liquid biopsies detect all relevant mutations present in the tumor or the relevant metastatic sites. A further aim of MORE is to study the reliability and relevance of ct-DNA in RCC patients.

## Conclusion

The MORE study will comprehensively gather whole-exome data of tumors and circulating DNA at baseline and under first and second progression together with all clinically relevant data of RCC patients under systemic treatment. It aims at a better biomarker understanding, better genomic subgrouping and the development of a more tailored therapy approach at choosing specific drugs for individual patients.

**Executive summary**

- Several target substances and immune checkpoint inhibitors are active and approved in renal cell carcinoma.
- Renal cell carcinoma is clonally heterogeneous.
- No molecular biomarkers predictive for any type of treatment are defined.
- Clonal evolution develops under therapy leading to progression.
- The described study aims at whole-exome sequencing of tumor and circulating tumor-DNA in blood to detect molecular biomarkers.
